# Editorial: Using physical & genomics markers for smart therapy via expert systems with computer learning

**DOI:** 10.3389/fgene.2023.1336399

**Published:** 2023-11-28

**Authors:** Yen-Wei Chu, Chi-Chang Chang

**Affiliations:** ^1^ Institute of Genomics and Bioinformatics, National Chung Hsing University, Taichung, Taiwan; ^2^ Institute of Molecular Biology, National Chung Hsing University, Taichung, Taiwan; ^3^ Agricultural Biotechnology Center, National Chung Hsing University, Taichung, Taiwan; ^4^ Ph. D. Program in Translational Medicine, National Chung Hsing University, Taichung, Taiwan; ^5^ Rong Hsing Research Center for Translational Medicine, Taichung, Taiwan; ^6^ Ph. D Program in Medical Biotechnology, National Chung Hsing University, Taichung, Taiwan; ^7^ School of Medical Informatics, Chung Shan Medical University & IT Office, Chung Shan Medical University Hospital, Taichung City, Taiwan; ^8^ Department of Information Management, Ming Chuan University, Taoyuan City, Taiwan

**Keywords:** predictive modeling, cancer biomarkers, machine learning, whole-evidence, cancer stem cells

Cancer diagnosis, prognosis, and treatment stand as pivotal factors in enhancing patient outcomes. Recent advances in AI, deep learning, and genomics are reshaping cancer research (Bhinder et al., 2021; Luchini et al., 2022). The integration of physical and genomics markers is essential for smart therapy. This is achieved through expert systems with computer learning (Chen et al., 2022; Deng et al., 2022; Ma et al., 2022; Chen et al., 2023; Sidorenkov et al., 2023).

Research in this domain has spotlighted predictive modeling and cancer biomarkers, demonstrating the application of machine learning in predicting malignancy and metastasis across various cancers, including lung and colorectal cancers (Ma et al., 2022; Wang et al., 2022; Mu et al., 2023). Furthermore, the emergence of novel combined models, integrating deep learning-pathomics and radiomics, holds promise in predicting postoperative outcomes in cancer patients (Wang et al., 2022).

Machine learning and whole-evidence analysis have facilitated comprehensive evaluation in cancer research. Studies focusing on lung cancer screening and hepatocellular carcinoma prognosis have enhanced screening and prognostic accuracy (Deng et al., 2022; Sidorenkov et al., 2023). Additionally, deep learning-based systems have demonstrated expert-level accuracy in delineating head and neck lymph node levels, contributing to advancements in radiotherapy research (Weissmann et al., 2023).

The investigation of cancer stem cells and their involvement in tumorigenesis has gained significant attention. Studies in this field have delved into the exploration of prognostic long non-coding RNAs (lncRNAs) and the development of deep learning models for early cancer diagnosis. These efforts underscore the critical importance of integrating both physical and genomic markers in treatment planning (Al Mamun et al., 2021).

In a stride toward addressing technical challenges in single-cell RNA-Seq data analysis, Huang et al. pioneered a novel approach integrating low-rank matrix completion for missing value imputation. Their work not only enhanced the accuracy of intratumor heterogeneity analysis but also laid a foundation for precise interpretations within the intricate landscape of cancer biology.

A pivotal study by Zhou et al. illuminated the significance of an immune-related prognostic signature in predicting the clinical outcomes and tumor immunity of stomach adenocarcinomas (STAD). Their findings underscored the pivotal role of immune cell infiltration, unveiling distinctive clusters of “hot” and “cold” tumors with divergent clinical trajectories, while identifying key genes, such as PEG10, DKK1, and RGS1, as critical prognostic markers.

Genes exert a significant impact not only on cancer but also on the development of various other diseases. Chuang et al. delved into the genetic nuances of diabetic retinopathy, shedding light on the influence of genetic variants of the lncRNA LINC00673 on disease susceptibility. Their exploration unveiled associations between specific LINC00673 single nucleotide polymorphisms (SNPs) and the development of non-proliferative diabetic retinopathy (NPDR), providing crucial insights into the genetic underpinnings of this complex ocular condition.

In parallel, Hsieh and Li harnessed the potential of image recognition techniques to rectify data imbalances in genetic disease research, spotlighting the transformative role of Synthetic Minority Oversampling Technique (SMOTE) in restoring data integrity within human biobanks. Their study streamlined data processing and facilitated more robust analysis and interpretation of genetic datasets.

In summary, these groundbreaking studies demonstrate the transformative potential of integrating physical and genomic markers into intelligent therapy systems, fortified by cutting-edge computational technologies. By fostering a collaborative and interdisciplinary approach that promotes scientific excellence, societal inclusivity, and ethical healthcare practices, we pave the way for a future where personalized medicine is not just a vision but a tangible reality accessible to all.

## References

[B1] Al Mamun A. Tanvir R. B. Sobhan M. Mathee K. Narasimhan G. Holt G. E. (2021). Multi-run concrete autoencoder to identify prognostic lncRNAs for 12 cancers. Int. J. Mol. Sci. 22 (21), 11919. 10.3390/ijms222111919 34769351 PMC8584911

[B2] Bhinder B. Gilvary C. Madhukar N. S. Elemento O. (2021). Artificial intelligence in cancer research and precision medicine. Cancer Discov. 11 (4), 900–915. 10.1158/2159-8290.CD-21-0090 33811123 PMC8034385

[B3] Chen T. Hu L. Lu Q. Xiao F. Xu H. Li H. (2023). A computer-aided diagnosis system for brain tumors based on artificial intelligence algorithms. Front. Neurosci. 17, 1120781. 10.3389/fnins.2023.1120781 37483342 PMC10360168

[B4] Chen W. Gong M. Zhou D. Zhang L. Kong J. Jiang F. (2022). CT-based deep learning radiomics signature for the preoperative prediction of the muscle-invasive status of bladder cancer. Front. Oncol. 12, 1019749. 10.3389/fonc.2022.1019749 36544709 PMC9761839

[B5] Deng P. Z. Zhao B. G. Huang X. H. Xu T. F. Chen Z. J. Wei Q. F. (2022). Preoperative contrast-enhanced computed tomography-based radiomics model for overall survival prediction in hepatocellular carcinoma. World J. Gastroenterol. 28 (31), 4376–4389. 10.3748/wjg.v28.i31.4376 36159012 PMC9453776

[B6] Luchini C. Pea A. Scarpa A. (2022). Artificial intelligence in oncology: current applications and future perspectives. Br. J. Cancer 126 (1), 4–9. 10.1038/s41416-021-01633-1 34837074 PMC8727615

[B7] Ma M. Gan L. Liu Y. Jiang Y. Xin L. Liu Y. (2022). Radiomics features based on automatic segmented MRI images: prognostic biomarkers for triple-negative breast cancer treated with neoadjuvant chemotherapy. Eur. J. Radiol. 146, 110095. 10.1016/j.ejrad.2021.110095 34890936

[B8] Mu J. Kuang K. Ao M. Li W. Dai H. Ouyang Z. (2023). Deep learning predicts malignancy and metastasis of solid pulmonary nodules from CT scans. Front. Med. (Lausanne) 10, 1145846. 10.3389/fmed.2023.1145846 37275359 PMC10235703

[B9] Sidorenkov G. Stadhouders R. Jacobs C. Mohamed Hoesein F. A. A. Gietema H. A. Nackaerts K. (2023). Multi-source data approach for personalized outcome prediction in lung cancer screening: update from the NELSON trial. Eur. J. Epidemiol. 38 (4), 445–454. 10.1007/s10654-023-00975-9 36943671 PMC10082103

[B10] Wang R. Dai W. Gong J. Huang M. Hu T. Li H. (2022). Development of a novel combined nomogram model integrating deep learning-pathomics, radiomics and immunoscore to predict postoperative outcome of colorectal cancer lung metastasis patients. J. Hematol. Oncol. 15 (1), 11. 10.1186/s13045-022-01225-3 35073937 PMC8785554

[B11] Weissmann T. Huang Y. Fischer S. Roesch J. Mansoorian S. Ayala Gaona H. (2023). Deep learning for automatic head and neck lymph node level delineation provides expert-level accuracy. Front. Oncol. 13, 1115258. 10.3389/fonc.2023.1115258 36874135 PMC9978473

